# Percutaneous biopsy in radiological imaging of the breast

**DOI:** 10.1590/0100-3984.2016.49.2e3

**Published:** 2016

**Authors:** Ellyete de Oliveira Canella

**Affiliations:** 1 Member of the Mammography Committee and Full Member of the Colégio Brasileiro de Radiologia e Diagnóstico por Imagem (CBR). E-mail: ellyete@unisys.com.br.

The article "Diagnostic underestimation of atypical hyperplasia and ductal carcinoma in situ at percutaneous core needle and vacuum-assisted biopsies of the breast in a Brazilian reference institution"^(1)^, published in the previous issue of **Radiologia Brasileira**, was produced at a referral teaching institution and reflects the level of responsibility required in order to perform invasive procedures in clinical practice.

In 1989, Dr. Parker started performing percutaneous biopsy, using a core needle and an automatic deployment device, for the diagnosis of nonpalpable breast lesions, the detection of which was becoming increasingly more frequent after the advent of high-resolution mammography. In their first study of the topic, published in 1990, Parker et al. compared the performance of core biopsy with that of surgical biopsy, concluding that core biopsy represents a reliable, rapid, and affordable alternative that is well-tolerated by patients with minimum complications and a low risk of parenchymal scarring^(2)^. In 1995, Dr. Parker developed the vacuum-assisted breast biopsy technique (using the Mammotome^®^ equipment), to improve the accuracy of biopsies of microcalcifications in adipose breast tissue^(3)^. Core and vacuum-assisted biopsies were initially greeted with skepticism and even rejected by some medical professionals. Image-guided percutaneous biopsies, however, would bring changes to the field and gain broad acceptance for use in clinical assessments.

Although the demand for percutaneous biopsies is currently increasing, many problems arise, resulting in false-negatives, late or delayed diagnosis, and increases in cost. Among the most important problems are the following: a) unawareness of the technical criteria for indicating the procedure (on the part of the requesting physician), as well as of the criteria for recommending and selecting the best type of biopsy (core or vacuum-assisted) and guidance (stereotactic, ultrasound, or magnetic resonance imaging) for each lesion (on the part of the radiologist), given that an incorrect indication/choice generates technical difficulties and diagnostic errors, as well as resulting in inefficient allocation of resources^(4)^; b) failure to standardize practices after a benign histopathological result, resulting in patients who should be merely observed undergoing surgery, and vice-versa; c) lack of technique and tactics in carrying out the procedure, including the incorrect choice of access and faulty spatial reasoning; d) lack of appropriate documentation of the procedure-the report, the record of the lesion before and after the biopsy, and X-rays of the biopsy fragments (if the lesion has microcalcifications)-leaving the radiologist vulnerable from a legal aspect^(5)^; e) lack of auditing of the results, making it impossible to evaluate the performance and identify errors to be corrected^(6-8)^. Items a), b) and c) are certainly the result of gaps in the education of the radiologist. It is extremely important that radiologists who work with breast imaging procedures frequent institutions that specialize in and are dedicated to specific training. Item d) illustrates the distortion in our model of care, in which low rates of pay by the medical insurance companies leads to an excessive number of exams per hour. That problem can be solved only by presenting a united front within the Brazilian College of Radiology and Diagnostic Imaging, because individually we are impotent. Item e) illustrates the absence of an auditing culture. There is no interest in the matter, and radiologists do not receive specific training in that. In addition, it is difficult to obtain and correlate results in day-to-day practice, because patients are lost in the system.

In conclusion, the considerations above are points for reflection. The ultimate goal is to improve the experience of patients subjected to breast biopsy.
